# Matrix metalloproteinases and tissue inhibitors of metalloproteinases in the aqueous humour of patients with primary angle closure glaucoma - a quantitative study

**DOI:** 10.1186/1471-2415-14-33

**Published:** 2014-03-24

**Authors:** Angeline DC Nga, Soon-Lek Yap, Amir Samsudin, Puteri S Abdul-Rahman, Onn H Hashim, Zahari Mimiwati

**Affiliations:** 1 Department of Ophthalmology, Faculty of Medicine, University Malaya, Kuala Lumpur 50603, Malaysia; 2 Department of Molecular Medicine, Faculty of Medicine, University Malaya, Kuala Lumpur 50603, Malaysia

**Keywords:** Matrix metalloproteinases, Tissue inhibitors of metalloproteinases, Aqueous humour, Primary angle-closure glaucoma, Primary open-angle glaucoma

## Abstract

**Background:**

Altered levels of specific matrix metalloproteinases (MMPs) and tissue inhibitors of metalloproteinases (TIMPs) in the aqueous humour of primary open-angle glaucoma (POAG) eyes have been described. In this study, levels of specific MMPs and TIMPs in the aqueous humour of primary angle-closure glaucoma (PACG) eyes were measured and compared with those of POAG as well as non-glaucoma control eyes.

**Methods:**

Aqueous humour from 16 PACG, 28 POAG and 27 control eyes were sampled during intraocular surgery. Levels of total protein, MMP-2, MMP-3, TIMP-1 and TIMP-2 were quantified by protein assay and enzyme immunoassay.

**Results:**

Total protein levels were significantly higher in PACG (0.426 ± 0.126 mg/ml, p = 0.043) and POAG (0.578 ± 0.360 mg/ml, p = 0.007) compared to controls (0.292 ± 0.192 mg/ml). The difference between PACG and POAG was not significant (p = 0.158). MMP-2 was significantly higher in PACG (p = 0.032) and POAG (p < 0.001) compared to controls. The difference between PACG and POAG was also not significant (p = 0.133). MMP-3 was significantly higher in POAG compared to controls (p = 0.002) and PACG (p = 0.029). The difference between PACG and controls was not significant (p = 0.962). TIMP-1 was significantly higher in PACG (p = 0.049) and POAG (p = 0.010) compared to controls. The difference between PACG and POAG was also not significant (p = 0.961). TIMP-2 was significantly higher in POAG (p = 0.004) compared to controls. The difference between PACG and either controls or POAG was not significant (p > 0.05). Although not statistically significant (p > 0.05), the MMP-2/TIMP-2 ratio was highest in PACG (2.83 ± 7.40), followed by POAG (1.38 ± 1.55) and controls (1.34 ± 3.05). Similarly, the MMP-2/TIMP-1 ratio was highest in PACG (1.50 ± 1.69), followed by POAG (1.40 ± 0.77) and controls (1.15 ± 0.92). The MMP-2 + MMP-3/TIMP-1 + TIMP-2 ratio was higher in PACG (0.83 ± 0.80) and POAG (0.82 ± 0.53) compared to controls (0.70 ± 0.63). In both POAG and PACG, there were no significant differences in the levels of total protein, MMP-2, MMP-3, TIMP-1 and TIMP-2 between patients on prostaglandin analogues and those not.

**Conclusion:**

We found altered levels of MMPs and TIMPs as well as imbalance of MMP:TIMP ratios in the aqueous humour of PACG eyes that were different from POAG and non-glaucoma control eyes.

## Background

The role of matrix metalloproteinases (MMPs) in degrading and remodelling extracellular matrix (ECM) is well documented [1,2]. In the eye, abnormal expression of MMPs has been implicated in corneal wound healing [3], pterygia formation [4], proliferative diabetic retinopathy [5] and age-related macular degeneration [6]. In relation to glaucoma, there is increasing awareness of their implications in the pathogenesis of primary open angle glaucoma (POAG) as well as uveitic and pseudoexfoliative glaucoma [2,7-10], where enhanced presence and activity of MMPs have been reported in the trabecular meshwork, Schlemm’s canal and aqueous humour of these diseased eyes.

MMPs are irreversibly inhibited in a 1:1 molar ratio by their specific endogenous tissue inhibitors of metalloproteinases (TIMPs) and both are normally present in balanced amounts [2,7-10]. The authors then suggest that any imbalance between MMPs and TIMPs may result in a change in ECM accumulation, which together with impaired trabecular meshwork matrix turnover causes increased aqueous humour outflow resistance and intraocular pressure (IOP). This provides an explanation of how altered levels of MMPs and TIMPs may upset the regulation of aqueous humour outflow facility, which may eventually lead to raised IOP and glaucoma.

Due to the high number of primary angle closure glaucoma (PACG) cases in our population, and its nature of being more visually destructive and difficult to manage than POAG, we were determined to find out if MMPs and TIMPs were present in balanced amounts in these cases. We searched the literature and found scarce information on eyes with PACG. In this study, we sought to find out if there were any similarities or differences in the levels of MMPs and TIMPs in PACG eyes, compared to POAG and control eyes.

Prostaglandin analogues are used in the treatment of glaucoma to reduce IOP. They increase MMP release by the ciliary smooth muscle cells, which leads to an increase in uveoscleral outflow [11,12]. We also looked into the effect of prostaglandin analogue use on the levels of total proteins, MMP-2, -3, TIMP-1 and -2 in our separate POAG and PACG groups.

## Methods

### Study design

This was a cross-sectional study involving patients undergoing elective cataract or combination cataract and trabeculectomy surgery at the University of Malaya Medical Centre, Kuala Lumpur. Ethical approval was obtained from the Medical Ethics Committee of the centre prior to commencement of the study. All patients signed a statement of informed consent in compliance with the tenets of the Declaration of Helsinki for experiments involving human tissue. POAG was diagnosed based on the guidelines suggested by Foster *et al.*[13], specifically when there was glaucomatous optic nerve head damage (defined by the presence of neuroretinal rim loss of the optic nerve head and vertical cup-disc ratio of 0.7 or greater) with corresponding visual field loss. Eyes with PACG had additional gonioscopic findings of angle closure (present when the trabecular meshwork was not visualised in at least three quadrants on non-indentation gonioscopy, with or without the presence of peripheral anterior synechiae). Diagnoses were confirmed by two fellowship-trained glaucoma specialists (AS and ZM).

The patients were on different numbers and combinations of anti-glaucoma medications, including prostaglandin analogues, beta blockers, carbonic anhydrase inhibitors and alpha-adrenergic agonists. Those who had previous surgical procedures e.g. trabeculectomies as well as those with uveitis, diabetes mellitus and connective tissue disease were excluded. Additionally, those who had peripheral iridotomies or acute angle closure attacks in the preceding 1-year were also excluded. Patients with senile cataract with no evidence of glaucoma served as the control group. All samples were from different individuals.

### Participants

A total of 33 (46.5%) male and 38 (53.5%) female patients participated in the study. Gender difference in the whole group as well as in PACG, POAG and control subgroups were not statistically significant (p > 0.05) from Mann–Whitney tests. Approximately 80–100 μl of aqueous humour was aspirated using a tuberculin syringe through an anterior chamber paracentesis at the start of the surgical procedure. Care was taken to avoid any contact with the inner structures of the eye and contamination with blood. The samples were then transferred into micro-centrifuge tubes, frozen in liquid nitrogen, stored at -80°C and thawed just before analysis.

### Estimation of protein, MMPs and TIMPs

Total aqueous humour protein levels were determined by a commercially-available Pierce Bicinchoninic Acid Protein Assay Kit (Thermo Scientific, IL, USA) with bovine serum albumin as the standard. MMP-2, MMP-3, TIMP-1 and TIMP-2 concentrations in the aqueous humour were measured with commercially-available 96-well enzyme immunoassay kits (Calbiochem, EMD Biosciences, Inc., Germany). Assays were performed in duplicate and a standard curve was plotted for each set. The MMP-2 assay detected total MMP-1 (proMMP-2, active MMP-2 and MMP-2:TIMP-2) complexes with a sensitivity of 0.1 ng/ml while the MMP-3 assay detected solely proMMP-3 with a sensitivity of 0.05 ng/ml. The total TIMP-1 and TIMP-2 assays had sensitivities of 0.0096 ng/ml and 3 ng/ml respectively.

### Statistical analysis

Most of the data did not show normal distributions when analysed using the One-Sample Kolmogorov-Smirnov test. The non-parametric Kruskal-Wallis test was thus used to compare the levels of total protein, MMP-2, MMP-3, TIMP-1 and TIMP-2 between the three groups. This was then followed by Dunn’s post-test to determine the individual differences between the groups. p < 0.05 was considered statistically significant.

## Results

**Figure 1:**
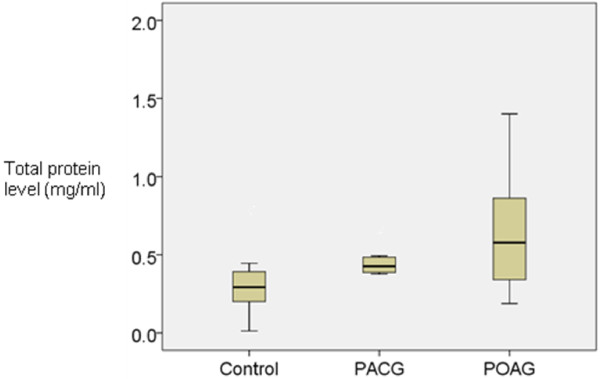
**Boxplot showing mean and distribution of total protein in the aqueous humour of control, PACG and POAG eyes.** The means for the control, PACG and POAG groups were 0.292, 0.426 and 0.578 mg/ml respectively. The levels were significantly higher in the PACG and POAG eyes compared to controls (p = 0.043 and 0.007 respectively).

Table 1 presents the demography, pre-operative IOP and number of medications of the control, PACG and POAG subjects. The total protein content in the aqueous humour of control, PACG and POAG eyes were 0.292 ± 0.192 mg/ml, 0.426 ± 0.126 mg/ml and 0.578 ± 0.360 mg/ml respectively (mean ± SD, Figure 1). The levels were significantly higher in the PACG and POAG eyes compared to the control eyes (p = 0.043 and 0.007 respectively). Although the mean was lower in the PACG eyes compared to the POAG eyes, the difference was not significant (p = 0.158).

**Table 1:** Demography, preoperative IOP and medications of subjects

**Characteristics**	**Control**	**PACG**	**POAG**
n	27 (38.0%)	16 (22.5%)	28 (39.5%)
Gender (male/female)	13/14	5/11	15/13
Age (years)	66.8 ± 8.3	65.5 ± 9.4	68.4 ± 13.1
Preoperative IOP (mmHg)	14.3 ± 2.7	17.3 ± 6.0	18.4 ± 4.2
IOP medications (units)	0	2.00 ± 1.03	2.14 ± 1.04

Age and IOP data shown as mean ± standard deviation.

**Figure 2:**
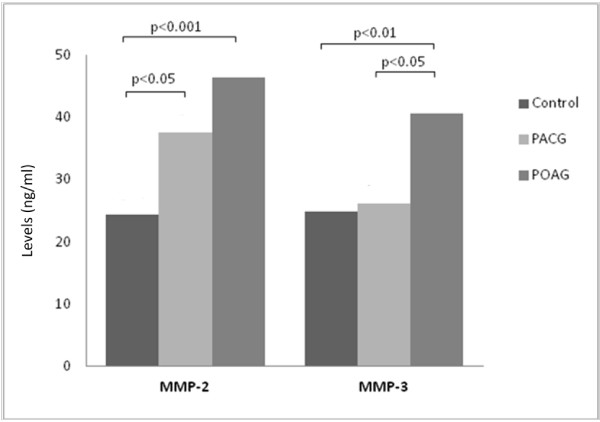
**Levels of MMPs in aqueous humour.** MMP-2 levels were significantly higher in the PACG and POAG groups compared to the control group. The difference was not significant between the POAG and PACG groups. MMP-3 levels were significantly higher in the POAG group compared to the control and PACG groups. The difference was not significant between the PACG and control groups.

MMP-2 levels were significantly higher in the PACG and POAG groups compared to the control group (p = 0.032 and <0.001 respectively, Figure 2). Although the mean was higher in the POAG group compared to the PACG group, the difference was not significant (p = 0.133). The level of MMP-3 was significantly higher in the POAG group compared to control (p = 0.002) and PACG (p = 0.029) groups (Figure 2). No significant difference was noted between the PACG and control groups (p = 0.962).

**Figure 3:**
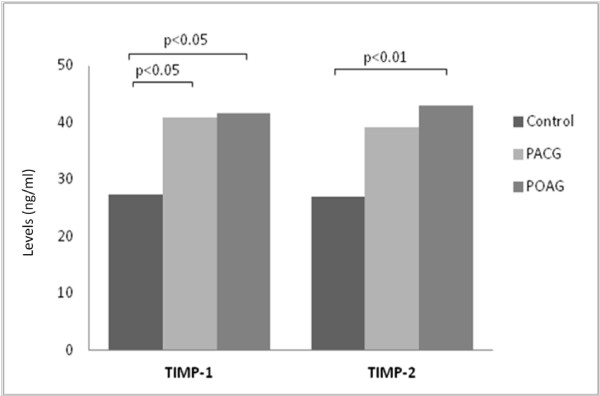
**Levels of TIMPs in aqueous humour.** TIMP-1 levels were significantly higher in PACG and POAG eyes compared to controls. No significant difference was noted between PACG and POAG eyes. TIMP-2 levels were significantly higher in POAG eyes compared to controls. There was no significant difference between PACG and either control or POAG eyes.

TIMP-1 levels were significantly higher in PACG (p = 0.049) and POAG (p = 0.010) eyes compared to controls (Figure 3). No significant difference was noted between PACG and POAG eyes (p = 0.961). TIMP-2 levels were significantly higher in POAG eyes compared to controls (p = 0.004). There was no significant difference between PACG and either control or POAG eyes (both p > 0.05).

When comparing various ratios of MMP to TIMP, the results were all not statistically significant (p > 0.05). However, the MMP-2/TIMP-2 ratio was highest in PACG (2.83 ± 7.40), followed by POAG (1.38 ± 1.55) and control eyes (1.34 ± 3.05) [Table 2]. A similar pattern was seen in the ratio of MMP-2/TIMP-1, which was highest in PACG (1.50 ± 1.69), followed by POAG (1.40 ± 0.77) and control eyes (1.15 ± 0.92). When ratios of the sum of MMPs-2 and -3/TIMPs-1 and -2 were calculated, the values were higher in the PACG (0.83 ± 0.80) and POAG (0.82 ± 0.53) groups compared to controls (0.70 ± 0.63).

**Table 2:** Ratios of MMP to TIMP

**Diagnosis**	**MMP-2/TIMP-2**	**MMP-2/TIMP-1**	**MMP-2 + 3 to TIMP-1 + 2**
Control	1.34 ± 3.05	1.15 ± 0.92	0.70 ± 0.63
PACG	2.83 ± 7.40	1.50 ± 1.69	0.83 ± 0.80
POAG	1.38 ± 1.55	1.40 ± 0.77	0.82 ± 0.53
P-value	0.732	0.309	0.372

No significant differences between control/PACG/POAG groups for all combinations on Kruskal-Wallis tests.

Total protein, MMP-2, MMP-3, TIMP-1 and TIMP-2 levels were compared between patients on prostaglandin analogues and those who were not, in both POAG and PACG groups (Table 3). In the POAG patients, there were no significant differences in the levels of total protein, MMP-2, MMP-3, TIMP-1 and TIMP -2 between those who were on prostaglandin analogues and those who were not. Similarly, in the PACG patients there were no significant differences in the levels of total protein, MMP-2, MMP-3, TIMP-1 and TIMP-2 between patients who were on prostaglandin analogues and those who were not.

**Table 3:** Comparison between prostaglandin analogue use and none, in both POAG and PACG groups

	**Prostaglandin analogue use**	**No prostaglandin analogue use**	**P-value**
**POAG**			
n	17	11	
Total protein (mg/ml)	0.67 ± 0.36	0.61 ± 0.49	0.404
MMP2 (ng/ml)	37.84 ± 23.53	32.08 ± 17.36	0.689
MMP3 (ng/ml)	14.88 ± 15.38	10.78 ± 16.21	0.063
TIMP1 (ng/ml)	32.18 ± 22.96	36.29 ± 41.44	0.742
TIMP2 (ng/ml)	61.56 ± 62.17	37.80 ± 29.16	0.375
**PACG**			
n	9	7	
Total protein (mg/ml)	0.47 ± 0.12	0.39 ± 0.13	0.899
MMP2 (ng/ml)	25.28 ± 12.73	29.07 ± 19.79	0.837
MMP3 (ng/ml)	20.02 ± 45.69	4.31 ± 2.48	0.692
TIMP1 (ng/ml)	43.81 ± 44.14	29.84 ± 14.77	0.681
TIMP2 (ng/ml)	47.52 ± 40.11	30.85 ± 22.33	0.408

## Discussion

Differences in aqueous humour MMP and TIMP levels between normal, PACG and POAG eyes were demonstrated in this study. In POAG eyes, the higher levels of both have been widely reported [2,7,9,10]. We found that our PACG eyes also had higher levels of total protein, MMP-2 and TIMP-1 compared to normal eyes. This implies a more active ECM degradation and remodelling process in the glaucomatous eyes. It is important to note that although the gelatinase MMP-2 is not a collagenase, it does have collagen type I degrading properties. Work investigating iris tissue composition in PACG eyes has been reported. He *et al.* observed an increase in mature collagen type I deposition in eyes that have suffered acute symptomatic episodes of angle closure, along with their contralateral fellow eyes [14]. However, they found that the levels were lower in chronic angle closure eyes. They went on to suggest that the development of PACG could be due to a difference in the ratio of collagen types I and III composition, which ultimately leads to a change in iris biomechanical properties and the development of PACG. The irides of PACG eyes are also known to have higher levels of SPARC (Secreted Protein, Acidic and Rich in Cysteine), which is a matricellular protein that is secreted by fibroblasts, endothelial cells and epithelial cells during an inflammatory response to facilitate matrix remodelling and tissue repair [15]. This protein is known to regulate the expression of several secreted ECM proteins and MMPs in certain cell types [16]. Also known as osteonectin and BM-40, SPARC binds to ECM proteins and cross-links collagen fibrils, particularly that of type I, to increase tensile strength and tissue rigidity. This change in iris rigidity influences its biomechanics, which in turn plays an important role in the development of angle closure mechanisms.

Although not statistically significant, the levels of total protein, MMP-2, TIMP-1 and TIMP-2 were lower in PACG eyes when compared to POAG eyes. The level of MMP-3 was significantly lower in PACG compared to in POAG, and comparable to the non-glaucoma controls. Seo *et al.* reported that PACG patients showed lower levels of fibrosis and MMP expression in Tenon’s tissue than POAG patients [17]. They suggested that the difference was due to involvement of distinctive pathological mechanisms. In POAG, the location of aqueous humour outflow obstruction is in the immediate vicinity of the trabecular meshwork and Schlemm’s canal; on the other hand, in PACG peripheral iris apposition blocks aqueous humour access to the trabecular meshwork. The authors remarked that these differences lead to the differential expression of the MMPs and ECM between the two conditions.

The evaluation of MMP/TIMP ratios was performed to look at possible imbalances in ECM modulation. Due to considerable inter-individual variations in MMP and TIMP, the results were not statistically significant. However, the MMP-2/TIMP-2 ratio was highest in PACG (2.83 ± 7.40), followed by POAG (1.38 ± 1.55) and controls (1.34 ± 3.05). A similar pattern was seen in the ratio of MMP-2/TIMP-1, which was highest in PACG (1.50 ± 1.69), followed by POAG (1.40 ± 0.77) and controls (1.15 ± 0.92). When ratios of the sum of MMP-2 and MMP-3 over the sum of TIMP-1 and TIMP-2 were calculated, they were higher in PACG (0.83 ± 0.80) and POAG (0.82 ± 0.53) compared to controls (0.70 ± 0.63). Schlotzer-Schrehardt *et al.* reported MMP-2/TIMP-2 ratios of 1.4 in POAG and 1.0 in control patients, and MMP-2 + MMP-3 to TIMP-1 + TIMP-2 ratios of 0.15 in POAG and 0.14 in control patients [7]. Fountoulakis *et al.* reported MMP-2/TIMP-2 ratios of 4.04 in POAG and 2.07 in control patients, and MMP-2/TIMP-1 ratios of 0.81 in POAG and 0.46 in control patients [10]. The exact measurements between studies vary perhaps due to the large inter-individual variations or differences in test kits but similar patterns can be seen. The ratios of MMP-2/TIMP-2 and MMP-2/TIMP-1 were higher in both POAG and PACG groups compared to controls, and higher in PACG than in POAG. When MMP-2 + MMP-3 and TIMP-1 + TIMP-2 were expressed as a ratio, TIMPs appeared in excess of MMPs. The ratios were also highest in the PACG group, followed by the POAG and control groups. We thus observed a change in the balance of MMPs and TIMPs in PACG and POAG eyes when compared to controls. This supports the current understanding that an imbalance between MMPs and their inhibitors is a key factor in abnormal matrix accumulation and causal involvement in the pathogenesis of POAG. Additionally, we conclude that this process occurs in PACG as well. From our findings and those by He *et al.* and Chua *et al.*[14,16], we believe that PACG eyes exhibit higher levels of MMPs and TIMPs due to abnormal collagen deposition. We look forward to further definitive studies which will shed more light into the pathogenesis of this blinding disease.

There were no significant differences in total protein, MMPs and TIMPs between our prostaglandin analogue treated eyes and those without, in both POAG and PACG groups. We are unclear about the cause of this but it may not be a representative picture as most of our glaucoma patients were on multiple anti-glaucoma agents, which could be a confounding factor modifying the levels of aqueous humour proteins, MMPs and TIMPs. This situation could have been prevented by analysing only those on monotherapy with prostaglandin analogues, but this was not performed as the number of patients was small (n = 2 in both POAG and PACG groups).

We recognise several other limitations of our study. We measured only MMP-2, MMP-3, TIMP-1 and TIMP-2. In future, we recommend measuring the levels of MMP1, MMP-8 and MMP-13 which are the specific collagenases that degrade collagen type I in particular. We also acknowledge the fact that previous acute angle closure attacks or laser peripheral iridotomy in PACG eyes could exert a local inflammatory effect and account for the observed differences in MMPs and TIMPs between the PACG and normal eyes. We did however exclude PACG cases with recent (within 1-year of aqueous sampling) peripheral iridotomies or attacks of acute angle closure for this study. Finally, we also included patients with systemic hypertension. This condition is associated with higher levels of circulating plasma MMP-2, MMP-9 and TIMP-1, which may also have affected our measurements [18].

## Conclusion

Our studies showed altered levels of MMPs and TIMPs, as well as unbalanced MMP:TIMP ratios in PACG eyes that were slightly different from those of POAG. These changes could be due to different expression of MMPs and TIMPs, and subsequent differences in ECM remodelling in the two conditions.

## Competing interests

The authors declare that they have no competing interests.

## Authors’ contributions

ADCN and ZM conceived the study. ADCN conducted the bench experiments, analysed the data and drafted the manuscript. ZM, SLY and AS were involved in sample collection and data analysis. PSA coordinated the study and assisted in manuscript preparation. OHH critically revised the manuscript. All authors have read and approved the final manuscript.

## Pre-publication history

The pre-publication history for this paper can be accessed here:

http://www.biomedcentral.com/1471-2415/14/33/prepub
